# Latitude-based approach for detecting aberrations of hand, foot, and mouth disease epidemics

**DOI:** 10.1186/s12911-015-0236-5

**Published:** 2015-12-24

**Authors:** Jia-Hong Tang, Ta-Chien Chan, Mika Shigematsu, Jing-Shiang Hwang

**Affiliations:** 1 Institute of Statistical Science, Academia Sinica, Taipei, 115 Taiwan; 2 Research Center for Humanities and Social Sciences, Academia Sinica, Taipei, 115 Taiwan; 3 National Institute of Infectious Diseases, Tokyo, 162-8640 Japan; 4 Institute of Statistical Science, Academia Sinica, 128 Academia Road, Section 2, Taipei, Taiwan

**Keywords:** Latitude, Sentinel surveillance, Enterovirus, Japan, Aberration detection, Prediction

## Abstract

**Background:**

Epidemics of hand, foot and mouth disease (HFMD) among children in East Asia have been a serious annual public health problem. Previous studies in China and island-type territories in East Asia showed that the onset of HFMD epidemics evolved with increased latitude. Based on the natural characteristics of the epidemics, we developed regression models for issuing aberration alerts and predictions.

**Methods:**

HFMD sentinel surveillance data from 2008 to 2014 in Japan are used in this study, covering 365 weeks and 47 prefectures between 24 and 46° of north latitude. Average HFMD cases per sentinel are standardized as Z rates. We fit weekly Z rate differences between prefectures located in the south and north of a designated prefecture with linear regression models to detect the surging trend of the epidemic for the prefecture. We propose a rule for issuing an aberration alert determined by the strength of the upward trend of south–north Z rate differences in the previous few weeks. In addition to the warning, we predict a Z rate for the next week with a 95 % confidence interval.

**Results:**

We selected Tokyo and Kyoto for evaluating the proposed approach to aberration detection. Overall, the peaks of epidemics in Tokyo mostly occurred in weeks 28–31, later than in Kyoto, where the disease peaked in weeks 26–31. Positive south–north Z rate differences in both prefectures were clearly observed ahead of the HFMD epidemic cycles. Aberrations in the major epidemics of 2011 and 2013 were successfully detected weeks earlier. The prediction also provided accurate estimates of the epidemic’s trends.

**Conclusions:**

We have used only the latitude, one geographical feature affecting the spatiotemporal distribution of HFMD, to develop rules for early aberration detection and prediction. We have also demonstrated that the proposed rules performed well using real data in terms of accuracy and timeliness. Although our approach may provide helpful information for controlling epidemics and minimizing the impact of diseases, the performance could be further improved by including other influential meteorological factors in the proposed latitude-based approach, which is worth further investigation.

## Background

Epidemiological surveillance is a routine process of collection, analysis and dissemination of health data for public health purposes. One function of infectious disease surveillance is to detect aberrations at an early stage. Early warning of aberrations could improve the efficiency of control campaigns and facilitate preventative actions to halt the spread of infectious diseases, thus reducing their impact on the health system [1]. Furthermore, morbidity and mortality would be reduced through an earlier and more efficient public health response.

Hand, foot and mouth disease (HFMD), which often strikes children under five years old, is caused by multiple enterovirus serotypes, and usually leads to mild or moderate symptoms, with recovery in about three to six days without medication [2]. Treatment of HFMD is limited, as there is currently no effective antiviral drug or vaccine [3]. So far, preventive measures, such as avoiding direct contact with infectious patients, disinfection of contaminated environments, and good personal hygiene habits, represent the best options for controlling and preventing HFMD infection [4].

Historically, the occurrences of HFMD epidemics were sporadic and local, but this pattern changed in the late 1990's. Since then, medium- to large-scale epidemics have been continuously observed in the Asia-Pacific region, including Singapore [5], Malaysia [6], Hong Kong [7], Taiwan [8], Japan [9], and China [10, 11]. Severe or lethal complications, such as encephalitis, meningitis, pulmonary edema and myocarditis, in the course of enterovirus infections drew attention to these diseases. In Taiwan, the sentinel physicians reported 129,106 cases of HFMD in 1998 [12]. There were 405 patients with severe disease, most of whom were five years old or younger; severe disease was seen in all regions of the island. Complications included encephalitis, aseptic meningitis, pulmonary edema or hemorrhage, acute flaccid paralysis, and myocarditis. Seventy-eight patients died, 71 of whom (91 %) were five years of age or younger. Of the patients who died, 65 (83 %) had pulmonary edema or pulmonary hemorrhage. From 2000 to 2002, many cases with complications were reported in Japan. Cases with complications included 226 (0.10 %) of the total 216,154 reported HFMD cases which occurred in 2000, 32 (0.02 %) of a total of 134,927 reported HFMD cases in 2001, and 14 (0.01 %) of the total 97,870 reported HFMD cases in 2002 [13]. Although severe or lethal complications are rare, some of these HFMD epidemics had unusually high numbers of fatalities, and this generated much fear and anxiety in this region [14]. Therefore, controlling the HFMD epidemics has become an emerging public health problem in these countries.

Detecting infectious diseases’ aberrations at an early stage is crucial for swift implementation of control measures. HFMD epidemics exhibit a significant seasonal pattern, with a rapid onset in the spring or summer, a gradual decline after the peak, and a mild second wave in the fall. This pattern has been observed not only in Asia, but also in European countries, such as Sweden, France and Hungary [15]. A bimodal seasonal pattern was reported in the United Kingdom, with peaks in summer, late autumn, and early winter [16]. In Finland, most HFMD cases were observed in autumn [17]. In an attempt to provide early warning for HFMD epidemics, a considerable amount of research has focused on developing statistical methods, including temporal, spatial, and spatiotemporal methods not only to contribute novel information but also to support aberration detection and management to identify aberrations in HFMD data accurately and quickly [18–21].

Meteorological factors have been recognized as spatial risk factors associated with HFMD occurrence. Weekly mean temperature and cumulated rainfall are significantly associated with HFMD incidence with a time lag of 1–2 weeks in Singapore [4]. A higher risk of transmission is associated with temperatures in the range of 70 °F to 80 °F, higher relative humidity, lower wind speed, more precipitation, and greater population density in China [22]. In Hong Kong, relative humidity, mean temperature, and difference in diurnal temperature were positively associated with HFMD consultation rates at a 2-week lag time [7]. In Japan, a study found that ambient temperature and relative humidity were associated with increased HFMD occurrence at a lag of 0–3 weeks [9]. In Taiwan, higher dew point, lower visibility, and lower wind speed were significantly associated with the rise of epidemics [23]. All these studies show that the dispersion of HFMD is sensitive to temperature variation.

In our previous study [23], we integrated the available surveillance and weather data in East Asia to elucidate possible spatiotemporal correlations between HFMD epidemics and the weather. The results revealed that latitude was the most important explanatory factor associated with the timing and amplitude of HFMD epidemics. In some population-based studies of HFMD in China, increasing amplitude of HFMD outbreaks was shown to accompany the increase of latitude in southern China [10, 24, 25]. Meteorological factors including higher dew point, lower visibility, and lower wind speed were significantly associated with the rise of epidemics. In addition, the temperature-related measurements also showed higher range in Japan than in other areas, which indicated the variations which occurred within Japan. Together with the decreasing trend of mean temperature from south to north, we inferred that latitude played an important role in change in temperature and would be associated with HFMD epidemics.

In this study, we propose a novel statistical approach based on linear regression models to detect the future trend of HFMD epidemics rather than detecting outbreaks of HFMD. The goals of this study were to characterize the influences of latitude variation on HFMD epidemics, to identify large epidemics of HFMD sufficiently early, allowing time for intervention, and to detect and predict HFMD epidemic trends with greater precision. The proposed approach would be used to facilitate efficient HFMD control.

## Methods

### Study area and surveillance data

**Fig. 1:**
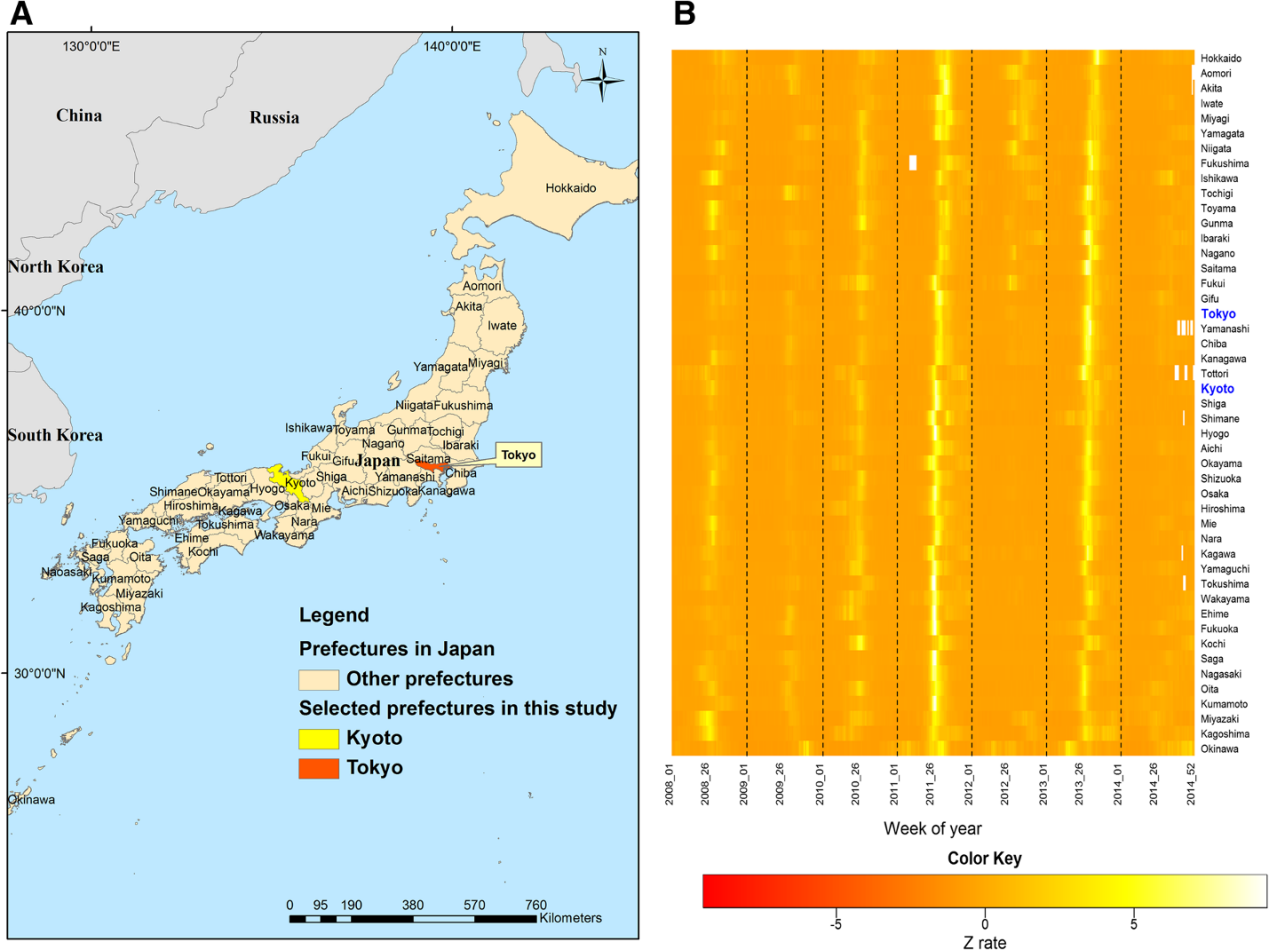
A map showing Japan's prefectures (**a**), and heat map of Z rate for HFMD by 47 prefectures of Japan (**b**). **a** A map showing Japan's prefectures. Japan is divided for administrative purposes into 47 prefectures stretching from Hokkaido in the north to Okinawa in the south. Tokyo is the capital of Japan, and is situated in the center of the Japanese archipelago. Kyoto, an ancient center of Japanese culture, is to the southwest of Tokyo. The original basemaps were downloaded from public available website, GADM database of Global Administrative Areas (http://www.gadm.org/) and further analyzed by the authors in this study. **b** The prefectures were ordered by latitude from southernmost (bottom) to northernmost (top). Note: The HFMD data of Fukushima in March of 2011 were not available due to the Great East Japan Earthquake, causing a white block on the heat map. The white blocks in Yamanashi, Tottori, Shimane, Kagawa and Tokushima are due to missing values

Japan is an archipelago nation in East Asia comprising four major islands and many small islands extending along the Pacific coast of Asia. It lies between 24° to 46° north latitude and 123° to 146° east longitude (Fig. 1a). There are 47 prefectures (local government administrative divisions). Japan lies mainly in the temperate zone, and is characterized by four distinct seasons.

The prefecture-level HFMD surveillance data from Japan, combined with latitudes of all Japanese prefectures, were used in this study. In Japan, infectious disease surveillance is designated as one of the important components for disease control, and its sentinel surveillance program was revised in 1999 to combine with the national notifiable diseases program, and incorporated into the national epidemiological surveillance infectious diseases (NEISD). The NESID in Japan, which was started in July 1981, is organized by the Ministry of Health, Labour and Welfare (MHLW), and encompasses the sentinel surveillance system for HFMD. NESID guidelines specify the method for selecting sentinel medical institutions [26, 27]. According to the guidelines, prefectural governments select sentinels as randomly as possible, and the numbers of sentinels per district public health center coverage area are determined in proportion to the population of the area in order to adequately assess any HFMD epidemic. HFMD, one of the sentinel reporting diseases in Japan, should be reported weekly by designated sentinels rather than reported immediately by all physicians; data are displayed by weekly reported number per sentinel. The designated sentinels send weekly HFMD data to the district health center on Tuesday of the next week. The health centers tabulate the district data and send it to the local health department on Wednesday. The weekly data are forwarded to MHLW by the local health departments the next day [26, 27].

In this study, latitudes of all Japanese prefectures, which were determined by the geographical center of each prefecture, and prefecture-level data from HFMD cases in Japan were collected online during the period from the 1^st^ week of 2008 to the 52^nd^ week of 2014 (a total of 365 weeks), from the National Institute of Infectious Diseases (NIID). These data are available at http://idsc.nih.go.jp. The HFMD dataset comprises weekly reported cases and cases per sentinel to provide an understanding of the epidemic situation and disease trends in different prefectures. To reflect the relative amplitude and severity of HFMD epidemics for each prefecture, we standardized the reported cases per sentinel separately in each prefecture during the study period, which are called Z rates in this study. The formula for the Z rate calculation is as follows:

$$ {Z}_{kt}=\left({S}_{kt}-{\mu}_k\right)/{\sigma}_k, $$

where *Z*_*kt*_ is the value of Z rate in prefecture *k* at week *t*, *S*_*kt*_ is the cases per sentinel in prefecture *k* at week *t*, and *μ*_*k*_ and *σ*_*k*_ are the mean and standard deviation of cases per sentinel in prefecture *k* during the study period. Thus, a positive Z rate indicates a datum above the mean of cases per sentinel, while a negative Z rate indicates a datum below the mean of cases per sentinel. The data we used were statistics publicly available online, and thus informed consent was not needed.

### Statistical method

With the assumption that HMFD epidemics spread from the south to the north, we propose three rules for estimating the trend of HFMD epidemics and predicting the cases per sentinel for the next week. First, we examine whether differences between the means of Z rates in areas south of a designated area and those north of it are increasing. If an increasing trend is identified, we activate the surveillance system and move to the second step to determine whether an aberration of HFMD cases is likely to occur in this area over the coming month. Finally, we predict the HFMD epidemic in the area one week ahead.

For convenience, all areas under study were sorted from southernmost to northernmost; for example, the latitude of the *k*^th^ area was the *k*^th^ lowest among all areas. To detect an unusual signal of HFMD activities in the *k*^th^ area, we calculate the difference between the means of Z rates in areas south of the *k*^th^ area and in areas north of the *k*^th^ area for the *t*^th^ week. The south–north Z rate difference is defined as

$$ {D}_{kt}=\frac{1}{m}{\displaystyle \sum_{j=k-m}^{k-1}{Z}_{jt}}-\frac{1}{n}{\displaystyle \sum_{j=k+1}^{k+n}{Z}_{jt}}, $$

where *m* and *n* represent the number of areas under study located to the south and to the north of the *k*^th^ area, respectively, with constraints of *m* < *k* and *n* ≤ *J* − *k*; *J* is the total number of areas under study; let *Z*_*jt*_ be the Z rate of the *t*^th^ week in the *j*^th^ area for *t* = 1, …, *T*, *j* = 1, …, *J*.

To detect HFMD epidemics future trend, we limit our focus on positive values of these differences. With the assumption that HMFD epidemics spread from the south to the north, positive *D*_*kt*_, …, *D*_*k*,*t* − *s*_ values in consecutive weeks indicate that the Z rates may have increased in the areas south of the *k*^th^ area in the past *s* weeks before the *t*^th^ week. When *D*_*kt*_ values have been increasing during the previous few weeks, we expect that the HMFD epidemics may spread from the south to the *k*^th^ area. If the area may be affected soon by the assumption that HMFD epidemics spread from the south to the north, the surveillance system should be activated. For determining whether an increasing Z rate in the coming weeks will occur for the *k*^th^ area, we propose a rule as follows:

#### Rule 1: Sending an activation signal

If *D*_*kt*_ > 0, ⋯, *D*_*k*,*t* − *s*_ > 0 and *D*_*k*,*t* − *s* − 1_ ≤ 0 for *s* > 1, we fit a linear regression model to these south–north Z rate differences, *D*_*k*,*t* − *i*_ = *μ*_*t*_ + *θ*_*t*_ × *i* + *ε*_*t* ‐ *i*_, for *i* = 0, 1, …, *s* and *s* ≤ 12.

If autocorrelation in the residuals has been shown to be present at week *t*, then an autoregressive model of order 1 is considered for the error term. That is, we assume *ε*_*t* − *i*_ = *φε*_*t* − *i* − 1_ + *w*_*t* − *i*_ and ${w}_{t-i}\overset{i.i.d.}{\sim }N\left(0,\ {\sigma}^2\right)$. The generalized least squares (GLS) regression analysis was used to estimate regression coefficients and their confidence limits.

The slope, *θ*_*t*_, represents the trend of the Z rate differences during the past *s* weeks. The 95 % lower bound of each *θ*_*t*_ was also calculated for judging whether the trend was significantly increasing during the few weeks before week *t*. Let $\widehat{\theta}{}_t$ and ${\widehat{\theta}}_t^L$ be, respectively, the estimate and the 95 % lower bound of the slope *θ*_*t*_ from the fitted model. We found that there was a considerable lag between the south–north Z rate differences and Z rates of a designated area. For most prefectures in Japan, the correlation coefficients between the south–north Z rate differences and Z rates reached statistically significant maximum values with a three-week or four-week lag. In a statistical sense, short and sporadic signals did not form a large-scale epidemic. Therefore, we consider the likelihood of HFMD aberration in areas south of the designated area to be increasing if the trend estimates were significant in three consecutive weeks. The designated area will very likely be hit in the coming weeks based on the assumption of HFMD epidemics spreading from the south. Therefore, we propose the first rule: send a signal to activate the surveillance system at the *t*^th^ week when we observe ${\widehat{\theta}}_t^L>0$, ${\widehat{\theta}}_{t-1}^L>0$ and ${\widehat{\theta}}_{t-2}^L>0$.

#### Rule 2: Issuing an aberration alert

When an activation signal appears in the *k*^th^ area, we then determine whether an aberration of HFMD cases is likely to occur in this area over the coming month (4 weeks). We assume that the Z rates of a designated area would be increasing in the coming month when an epidemic has started in the area. Suppose the aberration started at the *j*^th^ week; then we can use the slope estimate $\widehat{\pi}{}_j$ from the fitted linear regression model as a measure of intensity of the epidemic in the *k*^th^ area, *Z*_*k*, *j* + *u*_ = *α*_*j*_ + *π*_*j*_ × *u* + *ε*_*j* + *u*_, for *u* = 0, 1, 2, 3.

The larger the estimate $\widehat{\pi}{}_j$ is, the more sharply the Z rate will increase from the *j*^th^ week. However, at the *t*^th^ week, we have to wait three more weeks to obtain an estimate of *π*_*t*_ from the above linear regression model. We propose to use the relationship between two available trend estimates, $\widehat{\theta}{}_j$ and $\widehat{\pi}{}_j$, for *j* < *t* to construct a model for estimating *π*_*t*_ at the *t*^th^ week. Let *S* ⊂ {1, 2, …, *t*} be a set of indexes in which element *s* corresponds to the week when ${\widehat{\theta}}_{s-u}^L>0\ \mathrm{f}\mathrm{o}\mathrm{r}\ u=0,1,2$. We have also obtained ${\widehat{\pi}}_s$ for each *s* ∈ *S*\{*t* − 3, *t* − 2, *t* − 1, *t*}. We propose to first fit the linear regression model ${\widehat{\pi}}_s={\beta}_0+{\beta}_1{\widehat{\theta}}_s+{\varepsilon}_s\ \mathrm{f}\mathrm{o}\mathrm{r}\ s\in S\backslash \left\{t-3,t-2,t-1,t\right\}$.

Then we use the model estimates ${\widehat{\beta}}_0$ and ${\widehat{\beta}}_1$ to obtain the estimate of Z rate trend at the *t*^th^ week, i.e., ${\widehat{\pi}}_t={\widehat{\beta}}_0+{\widehat{\beta}}_1{\widehat{\theta}}_t$.

Our second rule is then proposed: issuing an HFMD epidemic alert in the *k*^th^ area at the *t*^th^ week when both $\widehat{\theta}{}_t$ > 0 and $\widehat{\pi}{}_t$ > 0.

In order to forecast the possibility and intensity of future epidemics, an epidemic monitoring indicator was set up. The slope estimate, $\widehat{\pi}{}_t$, which contains information about epidemic activity in the coming 4 weeks, is a suitable indicator for monitoring the trend of the HFMD epidemic. We categorized the epidemic trend of HFMD in the coming month as mild, moderate or strong based on the magnitude of this slope estimate. The slope can be interpreted as the percentage increase of Z rate per week. In this study, we choose 10 % and 30 % as cut-points to categorize the degree of severity of the designated area. The epidemic trend of HFMD in the coming month was categorized as mild if the value of percentage increase of Z rate in the coming month was below 10 %, moderate if it was between 10 % to 30 %, and strong if larger than 30 %.

Combined with Rule 1, we adopted a four-color gauge for visualizing the HFMD epidemic monitoring process, indicating the degree of severity of the epidemic, in which yellow represents an activation signal, while orange, red and purple stand for alerts of mild, moderate and strong epidemic trends in the coming month, respectively.

#### Rule 3: Predicting future epidemics

Since we have observed the influence of latitude variation on the temporal feature of HFMD epidemics, in which the annual timing of HFMD epidemics was earlier in southern than in northern areas, this relationship can also be used for improving prediction accuracy. A linear regression model was conducted to predict the HFMD epidemic one week ahead. However, the relevant data for constructing the predictive model are critical. Pearson’s correlation coefficient was used to identify areas in the south which are significantly associated with the designated area using the HFMD data of the past year. The HFMD data of the current year for those identified southern areas were then used for estimating regression parameters. Specifically, the regression model for prediction is

$$ {Z}_{h,t}={\gamma}_{0,t}+{\gamma}_{1,t}\ {Z}_{h,t-1}+{\varepsilon}_t,\ \mathrm{f}\mathrm{o}\mathrm{r}\kern0.5em 1\le h\le k, $$

where *h* includes the identified southern areas and the designated area. With the estimates of model parameters, the Z rate of the *k*^*th*^ area at week *t* + 1 could be predicted by

$$ {\widehat{Z}}_{k,\ t+1}={\widehat{\gamma}}_{0,t}+{\widehat{\gamma}}_{1,t}\ {Z}_{k,\ t}. $$

In the next section, we use HFMD data of the two selected prefectures in Japan, Tokyo and Kyoto, to illustrate the proposed approach. Tokyo, the capital of Japan, is the largest city in terms of population and is located roughly in the middle of the Japanese archipelago. Kyoto prefecture, the cultural center of Japan, is located southwest of Tokyo.

## Results

Japan has experienced nationwide epidemics of HFMD since the first HFMD case was diagnosed in Tokyo in 1963 [28]. The peak of the HFMD epidemic is usually seen in summer (June to August). However, epidemics may also occur in autumn and winter. A summary of annual data from 2008 to 2014 is shown in Table 1. The number of sentinels in Japan during 2008–2014 was about 3,100 for HFMD surveillance. There have been two large-scale HFMD epidemics since 2008, the first in 2011 (total 347,407 cases; 110.89 per sentinel) and the second in 2013 (total 303,339; 96.54 per sentinel). The year 2011 experienced the largest HFMD epidemic since the establishment of NESID.

**Table 1:** Annual data summary of HFMD sentinel surveillance, Japan, 2008–2014

Year	2008	2009	2010	2011	2012	2013	2014
Cumulative reported cases	145,185	68,578	151,021	347,407	72,822	303,339	83,683
Cumulative cases per sentinel	48.12	22.69	49.87	110.89	23.17	96.54	26.62
Numbers of sentinels	3017	3022	3028	3133	3143	3142	3144

Table 2 provides the prefecture-level HFMD data summary during 2008–2014. In 2011 and 2013, the two large-scale HFMD epidemic years, the weekly averages of cases per sentinel were 1–4 cases. The maximum values of cases per sentinel were between 4 to 42 in 2011 and 2013. The maximum value of cases per sentinel was 42.26 cases per sentinel in Saga prefecture in 2011. The weekly averages of cases per sentinel were less than one case for most prefectures in other years. The number of sentinels is determined in proportion to the population of a prefecture.

**Table 2:** Weekly prefecture-level HFMD data summary, Japan, 2008–2014

	2008			2009			2010			2011			2012			2013			2014				
Prefecture	Min Max Mean (per sentinel)			Min Max Mean (per sentinel)			Min Max Mean (per sentinel)			Min Max Mean (per sentinel)			Min Max Mean (per sentinel)			Min Max Mean (per sentinel)			Min Max Mean (per sentinel)			^a^Number of Sentinels	^b^Population (thousand)
Okinawa	0.06	1.68	0.63	0.00	4.97	0.70	0.06	1.32	0.33	0.18	5.97	1.86	0.03	2.79	0.92	0.18	5.24	1.39	0.00	4.38	1.19	34	1,421
Kagoshima	0.00	9.42	1.98	0.00	1.62	0.37	0.00	3.73	0.97	0.02	8.80	2.28	0.02	2.51	0.60	0.05	8.16	2.54	0.05	2.45	1.13	55	1,668
Miyazaki	0.03	14.89	3.06	0.00	3.50	0.82	0.11	6.25	1.99	0.14	14.28	2.97	0.00	5.17	1.20	0.00	7.08	2.16	0.33	4.94	1.72	36	1,114
Kumamoto	0.04	4.88	1.47	0.00	4.79	0.82	0.00	3.17	0.83	0.00	32.65	3.86	0.00	1.06	0.26	0.10	12.30	2.11	0.38	2.78	1.48	50	1,794
Oita	0.06	8.36	1.65	0.00	6.17	0.95	0.00	12.08	2.02	0.00	22.03	3.40	0.00	0.72	0.23	0.19	19.92	2.43	0.00	5.08	1.63	36	1,171
Nagasaki	0.05	4.73	1.33	0.00	3.77	0.64	0.02	1.30	0.45	0.02	19.32	2.94	0.00	0.43	0.12	0.00	13.57	2.09	0.07	1.59	0.63	45	1,386
Saga	0.00	4.96	1.27	0.00	6.13	1.36	0.00	1.74	0.45	0.00	42.26	4.63	0.00	0.61	0.09	0.05	12.30	2.52	0.04	3.65	1.07	23	835
Kochi	0.00	1.87	0.58	0.00	1.03	0.24	0.00	13.33	2.09	0.03	10.37	2.14	0.00	0.33	0.07	0.00	8.17	1.74	0.00	3.80	0.54	30	738
Fukuoka	0.03	3.21	0.83	0.03	8.63	1.46	0.08	4.15	0.94	0.03	40.96	4.41	0.00	1.08	0.08	0.40	11.12	2.46	0.23	5.18	1.38	121	5,091
Ehime	0.19	3.41	1.18	0.00	6.60	0.85	0.00	10.22	2.07	0.00	30.97	3.67	0.00	0.49	0.19	0.00	8.76	1.56	0.00	4.19	0.99	36	1,395
Wakayama	0.00	0.71	0.20	0.00	0.71	0.16	0.00	3.29	0.83	0.03	9.97	1.85	0.00	0.61	0.14	0.00	6.63	1.12	0.00	1.42	0.31	32	971
Tokushima	0.00	3.74	0.80	0.00	2.39	0.45	0.00	3.71	0.80	0.00	21.91	2.36	0.00	0.65	0.13	0.00	6.17	1.32	0.00	0.91	0.16	23	764
Yamaguchi	0.16	4.44	1.20	0.00	1.20	0.25	0.02	10.10	2.09	0.00	26.77	3.54	0.00	0.29	0.08	0.00	17.15	1.99	0.00	2.77	0.91	47	1,408
Kagawa	0.04	3.46	1.01	0.00	1.36	0.25	0.00	5.50	1.12	0.00	13.17	2.09	0.07	0.87	0.44	0.00	14.45	1.54	0.00	0.72	0.13	29	981
Nara	0.00	4.80	0.78	0.00	0.97	0.25	0.00	2.94	0.64	0.00	8.57	1.34	0.00	0.37	0.12	0.00	5.88	1.18	0.00	1.06	0.24	34	1,376
Mie	0.00	11.07	1.86	0.00	0.84	0.16	0.00	4.89	1.04	0.02	12.73	2.26	0.02	0.69	0.24	0.04	12.58	2.66	0.00	2.33	0.52	45	1,825
Hiroshima	0.10	3.11	0.89	0.00	0.82	0.24	0.00	3.40	1.19	0.03	12.51	2.43	0.00	0.22	0.08	0.08	11.42	1.84	0.00	1.28	0.35	72	2,833
Osaka	0.07	3.34	0.79	0.01	0.90	0.21	0.07	5.02	0.97	0.07	14.87	2.03	0.02	0.63	0.20	0.01	8.03	1.43	0.02	0.51	0.22	199	8,836
Shizuoka	0.00	8.76	1.34	0.01	1.33	0.25	0.00	8.19	1.19	0.00	14.19	2.16	0.00	0.52	0.14	0.00	12.01	2.14	0.00	0.75	0.20	89	3,705
Okayama	0.04	3.50	0.87	0.00	1.15	0.24	0.02	2.06	0.74	0.02	10.61	2.19	0.02	0.82	0.24	0.04	8.63	1.40	0.00	0.87	0.21	54	1,924
Aichi	0.07	4.06	0.96	0.02	0.86	0.20	0.03	3.88	0.84	0.04	10.87	2.29	0.01	0.33	0.11	0.01	11.10	1.65	0.03	1.71	0.51	182	7,455
Hyogo	0.04	3.88	0.87	0.01	1.16	0.21	0.05	7.20	1.34	0.03	26.38	2.84	0.01	0.28	0.10	0.00	9.69	1.45	0.01	0.65	0.24	128	5,541
Shimane	0.04	1.48	0.52	0.00	2.52	0.60	0.00	3.35	0.75	0.00	17.13	3.06	0.00	0.44	0.07	0.00	8.39	2.34	0.00	0.78	0.21	22	697
Shiga	0.00	3.06	0.65	0.00	2.00	0.41	0.00	7.38	1.22	0.06	20.34	3.08	0.00	1.22	0.31	0.00	7.22	1.79	0.00	0.66	0.30	32	1,416
Kyoto	0.05	4.45	0.89	0.00	0.92	0.20	0.00	3.85	0.83	0.00	13.42	1.74	0.00	0.49	0.17	0.00	5.27	1.26	0.00	0.96	0.28	74	2,610
Tottori	0.00	4.16	1.13	0.00	0.84	0.16	0.00	3.63	0.81	0.00	9.11	1.87	0.00	0.42	0.06	0.00	13.42	2.50	0.00	0.47	0.08	20	574
Kanagawa	0.00	5.89	1.08	0.01	2.56	0.47	0.04	3.75	0.67	0.01	10.20	1.80	0.01	1.23	0.46	0.04	11.43	1.75	0.00	1.98	0.40	204	9,096
Chiba	0.02	2.23	0.53	0.02	3.02	0.53	0.04	2.68	0.70	0.02	7.95	1.65	0.02	0.96	0.36	0.00	11.59	1.96	0.00	1.38	0.51	133	6,197
Yamanashi	0.00	0.58	0.14	0.00	1.29	0.29	0.00	1.17	0.30	0.00	11.00	1.42	0.00	0.71	0.20	0.00	17.92	2.44	0.00	1.13	0.23	24	841
Tokyo	0.01	2.04	0.59	0.03	1.98	0.44	0.05	4.69	0.82	0.02	11.07	1.84	0.01	1.38	0.40	0.05	15.75	2.21	0.01	1.55	0.45	258	13,390
Gifu	0.00	2.72	0.68	0.00	2.08	0.28	0.00	1.89	0.45	0.02	10.91	1.75	0.00	0.40	0.11	0.00	7.08	1.21	0.00	2.38	0.54	52	2,041
Fukui	0.00	2.82	0.61	0.00	1.73	0.36	0.09	8.91	2.60	0.14	16.23	2.32	0.09	9.86	1.60	0.00	11.64	2.01	0.00	2.36	0.44	22	790
Saitama	0.03	2.07	0.56	0.03	1.69	0.45	0.08	3.65	0.80	0.03	7.96	1.44	0.01	1.44	0.52	0.06	18.69	2.73	0.01	1.61	0.55	156	7,239
Nagano	0.02	8.40	1.45	0.00	1.26	0.22	0.00	3.16	0.70	0.00	9.04	1.50	0.02	4.87	0.63	0.00	14.25	1.96	0.00	1.35	0.41	53	2,109
Ibaraki	0.01	1.31	0.49	0.00	1.25	0.38	0.00	1.51	0.36	0.00	4.09	0.76	0.00	2.15	0.55	0.08	9.41	1.45	0.00	1.38	0.42	75	2,919
Gunma	0.02	6.37	1.01	0.00	0.66	0.18	0.02	7.90	1.25	0.00	5.18	1.04	0.00	1.48	0.36	0.02	7.37	1.31	0.00	1.19	0.36	59	1,976
Toyama	0.00	9.59	1.54	0.00	0.79	0.21	0.03	5.07	1.27	0.00	8.69	1.59	0.00	1.24	0.45	0.07	7.62	2.03	0.00	1.52	0.40	29	1,070
Tochigi	0.00	1.17	0.40	0.00	5.23	0.90	0.00	3.02	0.67	0.02	2.54	0.77	0.00	2.15	0.57	0.02	10.35	1.67	0.00	0.94	0.31	48	1,980
Ishikawa	0.00	11.41	1.97	0.00	0.93	0.20	0.00	6.79	1.35	0.00	12.03	1.91	0.03	2.14	0.85	0.10	7.62	1.62	0.03	6.62	1.32	29	1,156
Fukushima	0.00	1.60	0.44	0.00	0.92	0.29	0.02	4.42	0.93	0.00	5.63	1.49	0.00	2.58	1.01	0.04	10.04	1.49	0.00	1.13	0.32	45	1,935
Niigata	0.02	12.43	1.94	0.00	1.31	0.35	0.05	10.46	1.57	0.00	5.85	1.06	0.07	12.08	1.90	0.11	21.03	3.41	0.00	1.28	0.29	62	2,313
Yamagata	0.00	1.47	0.51	0.00	4.00	0.94	0.17	7.17	1.48	0.00	14.70	2.80	0.00	7.40	1.36	0.03	8.31	1.62	0.00	2.77	0.70	29	1,131
Miyagi	0.00	2.62	0.71	0.00	0.70	0.18	0.02	6.17	1.12	0.00	10.64	2.69	0.00	6.93	1.45	0.02	6.17	1.54	0.00	0.62	0.18	59	2,328
Iwate	0.00	1.25	0.36	0.03	1.28	0.40	0.03	3.13	0.88	0.00	14.03	3.09	0.00	4.65	1.18	0.00	7.26	1.28	0.00	1.88	0.32	40	1,284
Akita	0.00	1.83	0.53	0.00	4.97	0.74	0.00	1.69	0.48	0.00	16.17	2.05	0.00	3.60	1.04	0.00	7.71	1.07	0.00	0.50	0.10	34	1,037
Aomori	0.00	2.50	0.62	0.02	4.91	1.12	0.00	2.33	0.52	0.05	14.20	2.50	0.00	7.29	1.62	0.00	6.12	1.17	0.00	0.50	0.13	41	1,321
Hokkaido	0.00	3.48	0.72	0.00	1.27	0.24	0.01	2.93	0.82	0.03	5.91	1.30	0.06	3.62	0.69	0.01	12.73	1.56	0.01	1.31	0.50	143	5,400

*Notes*. ^a^The HFMD data of 2014 is interim, not final

^b^The official population estimates of Japan's prefectures are according to those reported by the Statistics Bureau of Japan (http://www.e-stat.go.jp/) as of October 1, 2014

To further explore the relationships of geographical locations of prefectures in Japan to features of HFMD epidemics, a heat map created using the gplot package in R software is provided in Fig. 1b. The heat map summarizes information on week of year in columns, and integrates prefecture-level HFMD Z rate data sets during the study period in rows. Larger values are represented by lighter color blocks and smaller values by darker color blocks. From bottom to top, prefectures in Japan were sorted by latitude from low to high. Lighter color blocks in each row indicated the timing of the HFMD peak period of each prefecture in Japan. The two brightest timing bands in Fig. 1b display two large-scale HFMD epidemics for 2011 and 2013, respectively. From bottom to top, the two brightest timing bands show that the HFMD peak time of each prefecture in Japan moved from left to right gradually. This phenomenon reveals the prefecture-level HFMD peak time in Japan moving in a south–north direction over time.

**Fig. 2:**
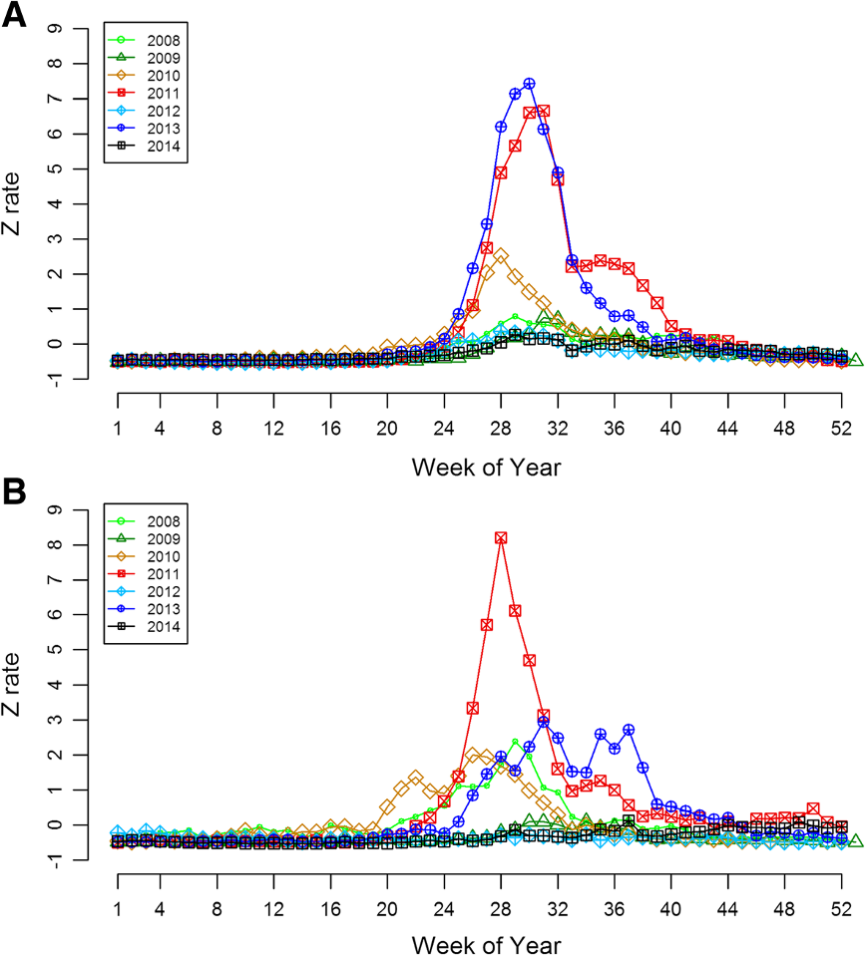
Weekly Z rates distribution of HFMD, Tokyo, 2008 to 2014 (**a**), and weekly Z rates distribution of HFMD, Kyoto, 2008 to 2014 (**b**). **a** The epidemic peaks in Tokyo mostly occurred in weeks 28–31 during the study period. **b** The epidemic peaks in Kyoto mostly occurred in weeks 26–31 during the study period

Figure 2a indicates that there have been three large HFMD epidemics in Tokyo during 2008–2014, the first in 2010, the second in 2011, and the last in 2013. Most HFMD epidemics in Tokyo have displayed a common trend of steady increase beginning in April or May, rapid increase during May or June, a peak from July to August, a quick decline in September, and finally, steady decrease until the next February. Figure 2b presents four large-scale epidemics in Kyoto during 2008–2014 which occurred in 2008, 2010, 2011 and 2013. The Z rates were low in January to March in Kyoto, then began to ascend starting in April, and a sharp increase appeared during June to July. A comparison between Figs. 2a and b reveals that the epidemic of 2013 was the largest one since 2008 in Tokyo, while the epidemic of 2011 was the largest one in Kyoto. Overall, the epidemic peaks in Tokyo mostly occurred in weeks 28–31, later than in Kyoto, where the peaks mostly occurred in weeks 26–31.

**Fig. 3:**
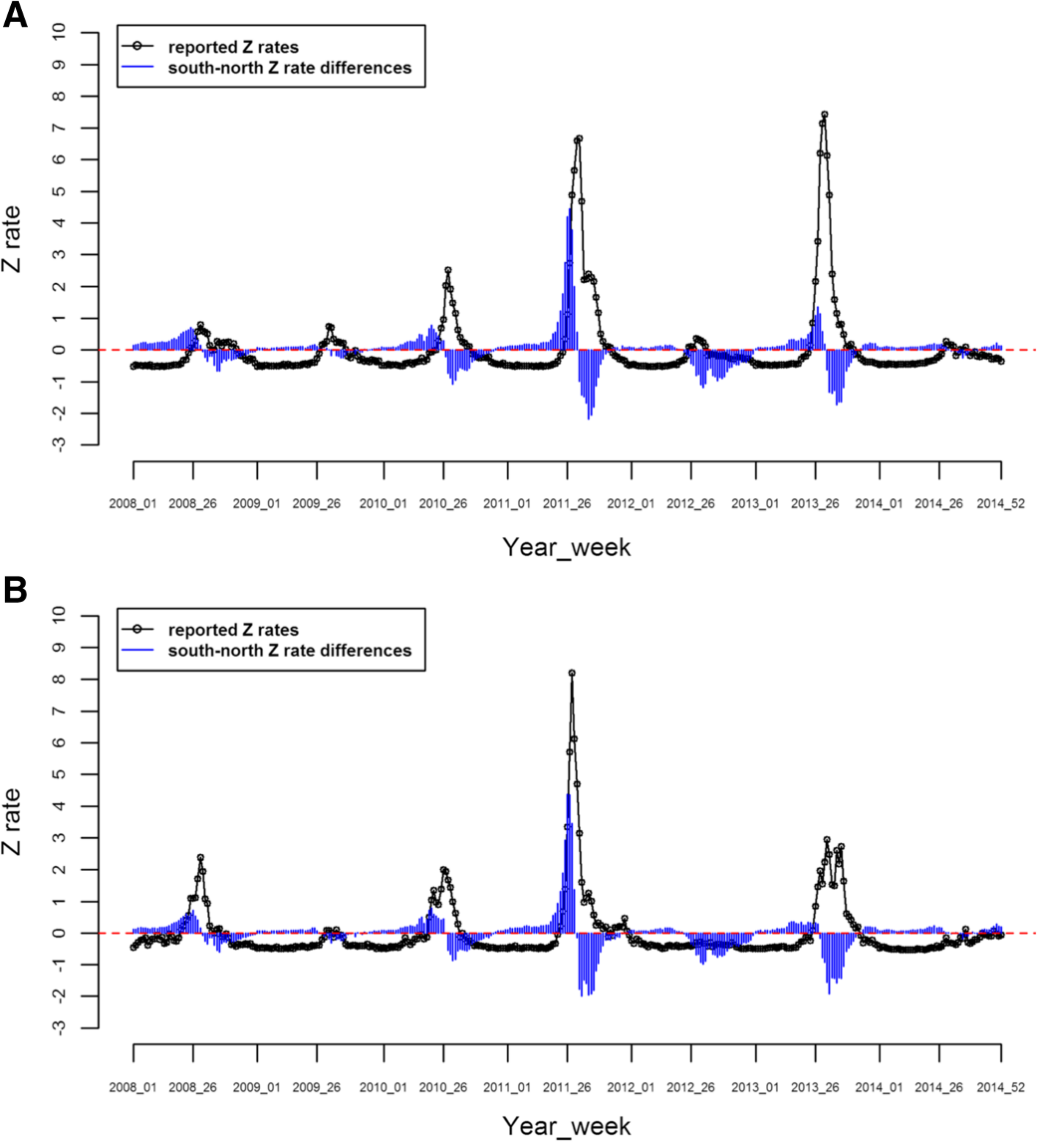
The south–north Z rate differences together with weekly Z rates, Tokyo, 2008–2014 (**a**), and the south–north Z rate differences together with weekly Z rates, Kyoto, 2008–2014 (**b**). **a** Blue lines represent the difference between the means of Z rates in areas south of Tokyo and in areas north of Tokyo for each week in the study period. **b** Blue lines represent the difference between the means of Z rates in areas south of Kyoto and in areas north of Kyoto for each week in the study period

The south–north Z rate differences of Tokyo and Kyoto are shown in Figs. 3a and b, together with their weekly Z rates. It is clear that the two weekly series had similar patterns and that the cycles of the south–north Z rate differences are ahead of the HFMD epidemic cycles in both figures. The peak of the south–north Z rate differences is much earlier than the peak of Z rates.

**Fig. 4:**
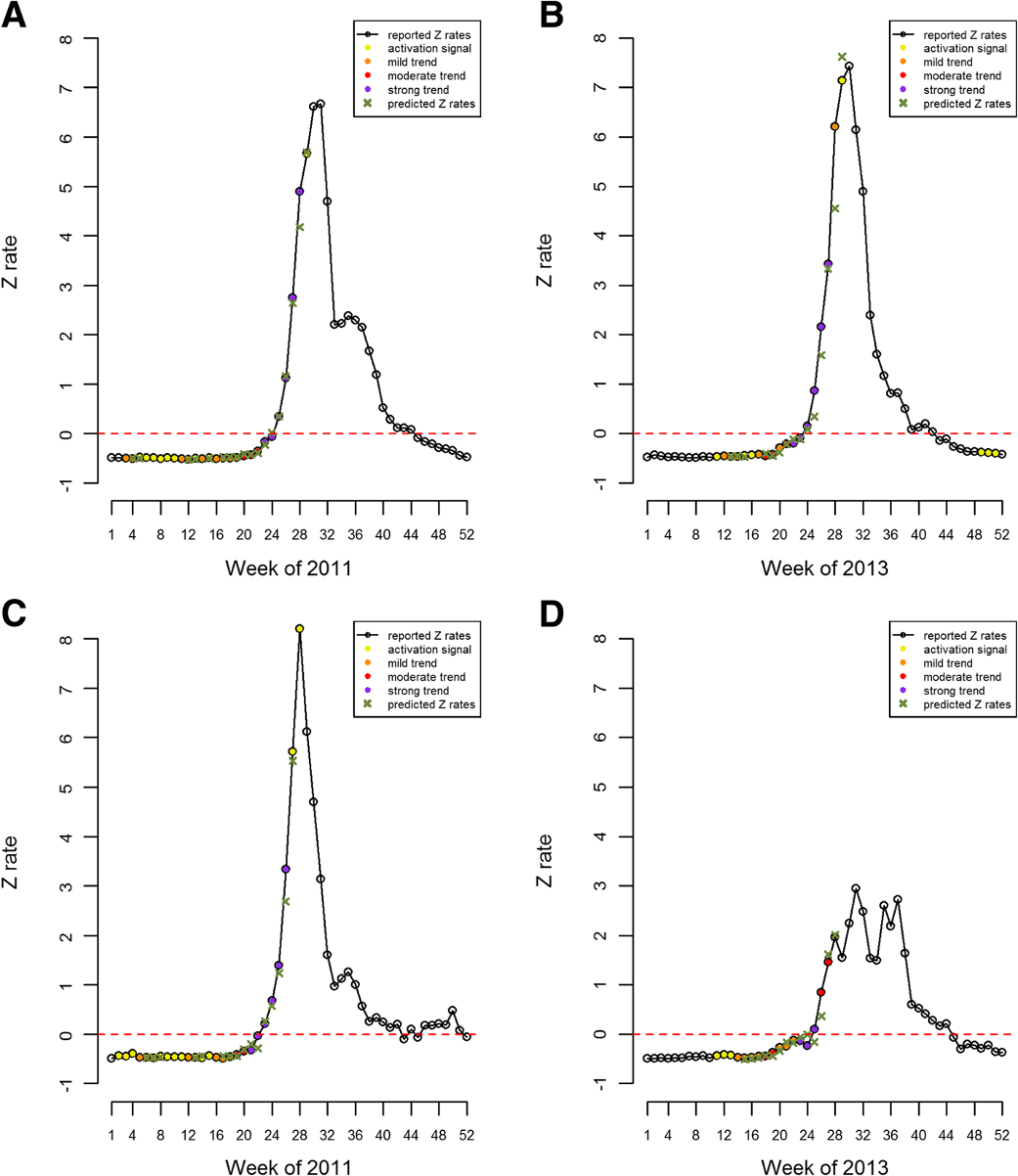
HFMD epidemic monitoring indicators, Tokyo, 2011 (**a**) and 2013 (**b**) and HFMD epidemic monitoring indicators, Kyoto, 2011 (**c**) and 2013 (**d**). The epidemic trend of HFMD in the coming weeks were categorized as mild (orange), moderate (red) and strong (purple). The yellow represents an activation signal

Figures 4a and b illustrate HFMD epidemic monitoring indicators in Tokyo and Kyoto in 2011 and 2013, respectively. The monitoring indicators gauge the epidemic trend of HFMD in the following weeks. In Fig. 4a, the monitoring indicators in Tokyo showed colors of activation or mild signals before week 20. Purple signals, indicating the momentum of the epidemic was strong, started to flash from week 23 to week 28, and the peak was reached at week 31 in 2011. Figure 4b reveals that the monitoring indicators in Tokyo began to send an activation signal at week 11, then alerts turned from mild to moderate; finally the monitoring indicators also registered a 6^th^ consecutive strong trend at week 27, and the peak was reached at week 30 in 2013. In Kyoto, the first alert, an activation signal, was issued at week 2, and the monitoring indicators flashed 6 consecutive purple signals starting from the 21^st^ week; the peak was reached at week 28 in 2011. The epidemic in Kyoto in 2013 was smaller but more irregular than the epidemic in 2011. Figure 4d shows that it seems to have two peaks in Kyoto in 2013. The monitoring indicators in Kyoto began to send an activation signal at week 11, and registered a third consecutive purple signal at the 25^th^ week in 2013. There were no alerts issued for the second peak in Kyoto in 2013. In Fig. 4, we can also observe that there are no alerts issued during the second half of 2011 and 2013 (the non-epidemic periods) except a total of 3 activation alerts in Tokyo in 2013.

Major epidemics during 2011 and 2013 in Tokyo and Kyoto were predicted and are shown for these two years separately in Tables 3 and 4. The predicted values of Z rates are converted to weekly cases per sentinel and listed in these two tables. Although most 95 % predicted intervals cover true values, the predictive model slightly underestimates weekly cases per sentinel during the peak weeks. The average absolute errors of predicted values for 2011 and 2013 in the two cities were 0.14, 0.49, 0.23 and 0.17 cases per sentinel, respectively. Figures 4a and b also clearly demonstrate the relationship between the true values and the predicted values. Overall, the predicted model provides effective prediction of HFMD epidemic trends.

**Table 3:** Weekly reported and predicted cases per sentinel of major HFMD epidemics, Tokyo, 2011 and 2013

Year	Week	Monitoring indicator	Reported	Predicted	95 % predicted interval		Absolute error
					Lower limit	Upper limit	
2011	17	mild	0.054	0.084	0.030	0.137	0.030
	18	mild	0.054	0.093	0.030	0.156	0.039
	19	mild	0.069	0.088	0.041	0.135	0.019
	20	moderate	0.122	0.181	0.059	0.303	0.059
	21	moderate	0.157	0.165	0.000^a^	0.341	0.008
	22	moderate	0.273	0.211	0.086	0.337	0.062
	23	strong	0.556	0.467	0.244	0.689	0.089
	24	strong	0.714	0.841	0.608	1.075	0.127
	25	strong	1.342	1.347	1.004	1.690	0.005
	26	strong	2.540	2.596	2.085	3.107	0.056
	27	strong	5.038	4.881	4.163	5.600	0.157
	28	strong	8.341	7.241	6.337	8.145	1.100
	29	activation	9.531	9.569	8.712	10.426	0.038
Average							0.138
2013	18	moderate	0.090	0.162	0.118	0.206	0.072
	19	moderate	0.130	0.103	0.052	0.155	0.027
	20	mild	0.330	0.198	0.147	0.248	0.132
	21	moderate	0.450	0.448	0.387	0.510	0.002
	22	strong	0.470	0.598	0.514	0.682	0.128
	23	strong	0.640	0.595	0.499	0.691	0.045
	24	strong	0.990	0.895	0.814	0.977	0.095
	25	strong	2.050	1.270	1.125	1.415	0.780
	26	strong	4.000	3.138	2.774	3.501	0.862
	27	strong	6.010	5.878	5.335	6.420	0.132
	28	mild	10.970	8.265	7.355	9.175	2.705
	29	activation	13.710	14.565	12.410	16.721	0.855
Average							0.486

^a^The negative value is replaced with zero

**Table 4:** Weekly reported and predicted cases per sentinel of major HFMD epidemics, Kyoto, 2011 and 2013

Year	Week	Monitoring indicator	Reported	Predicted	95 % predicted interval		Absolute error
					Lower limit	Upper limit	
2011	17	mild	0.000	0.073	0.011	0.135	0.073
	18	mild	0.054	0.068	−0.012	0.147	0.014
	19	moderate	0.122	0.085	0.038	0.132	0.037
	20	moderate	0.233	0.279	0.161	0.396	0.046
	21	strong	0.257	0.450	0.276	0.623	0.193
	22	strong	0.703	0.326	0.177	0.475	0.377
	23	strong	1.081	1.168	0.950	1.386	0.087
	24	strong	1.808	1.641	1.390	1.891	0.167
	25	strong	2.905	2.671	2.385	2.957	0.234
	26	strong	5.917	4.902	4.500	5.303	1.015
	27	activation	9.581	9.299	8.530	10.069	0.282
Average							0.230
2013	15	mild	0.000	0.000^a^	0.000^a^	0.063	0.000
	16	mild	0.010	0.000^a^	0.000^a^	0.029	0.010
	17	mild	0.040	0.000^a^	0.000^a^	0.065	0.040
	18	moderate	0.040	0.065	0.012	0.117	0.025
	19	moderate	0.160	0.050	0.000^a^	0.107	0.110
	20	mild	0.310	0.208	0.167	0.249	0.102
	21	mild	0.340	0.462	0.384	0.540	0.122
	22	mild	0.520	0.451	0.297	0.605	0.069
	23	strong	0.510	0.612	0.498	0.726	0.102
	24	strong	0.360	0.698	0.597	0.799	0.338
	25	strong	0.850	0.474	0.305	0.643	0.376
	26	moderate	1.970	1.258	1.050	1.466	0.712
	27	moderate	2.890	3.125	2.835	3.416	0.235
	28	--	3.660	3.747	3.462	4.031	0.087
Average							0.166

^a^The negative value is replaced with zero

--: Not available

## Discussion

Our study of the influence of latitude on the spatiotemporal characteristics of HFMD epidemics has yielded several notable findings. The two brightest timing bands in Fig. 1b reveal the influence of latitude variation on the spatiotemporal features of the HFMD epidemic, with the peak time moving in a south–north direction over time. The influence of latitude variation on the spatial spreading of HFMD is clear and provides an important basis for detecting HFMD epidemic trends in this study. In other words, the annual epidemic of HFMD started in the south and then gradually spread to the north. We adopt the correlation coefficient as the evaluation indicator to identify the relationship between the south–north Z rate differences and Z rates of a designated area in Rule 1. For most prefectures in Japan, the correlation coefficients between the south–north Z rate differences and Z rates reached statistically significant maximum values with a three-week or four-week lag. These lag values indicate that the south–north Z rate differences are ahead of the HFMD epidemic cycles and provide an important basis for Rule 1. A four-color gauge for the HFMD epidemic monitoring process, indicating the degree of severity of the epidemic, is provided in Rule 2. The monitoring results show that the proposed statistical approach, which takes into consideration the impact of latitude variation on HFMD epidemics, performed well in early aberration detection and predicting the epidemic trend. On the basis of the temporal feature of HFMD epidemics, this study also developed models for prediction of the activity of HFMD epidemics one week ahead, with an alert issued by the proposed aberration detection rules. For HFMD epidemics exhibiting annual variation, the predictive model is used to calculate a predicted value for the next week based on current year data.

Weekly prefecture-level data from 2008 to 2014 were adequate for exploring spatiotemporal trends of HFMD epidemics in Japan. When many spatial regions are under surveillance, aberration detection methods that contain spatial information may be more powerful, but they require an understanding of the nature of the spatial pattern, including how it changes over time. Zhuang et al. [25] extracted the spatial distribution of HFMD infections in China and found that regions with a higher monthly incidence rate of HFMD periodically shifted, following the pattern of south–north–south from March to December. In this study, only the south–north Z rate differences were used in the regression model to detect the spatial spreading of HFMD. Spatial autocorrelation may be simultaneously taken into account in the future work so as to faithfully determine the influence of latitude variation [29, 30].

The spatiotemporal characteristic of latitude was identified in this study. For more comprehensively and objectively understanding the influences of surrounding factors on spatiotemporal trends of HFMD epidemics, more factors with potential impact, such as population density, population flow, medical level, etc., should be taken into consideration in the spatiotemporal modeling for further study [30–32].

The idea of categorizing the epidemic trend of HFMD into three groups (mild, moderate and strong) was for convenience of distinguishing the different severity levels of the future HFMD epidemic trends. However, it is crucial to choose the cut-off points to determine which kind of aberration alert should be issued. In this study, the value of slope was expressed in terms of percentage increase to reflect the severity levels of the future epidemic trend in Japan. The relative amplitude and severity of HFMD epidemic vary according to different causative viruses and different geographical regions, so cut-off points of the slope estimates for determining these three categories may be chosen to suit local circumstances. In this study, we also tried to detect and predict HFMD epidemics trend by considering only the spatial differences and the timing of the aberrations. Although the predicted model fits the epidemic trend well, producing accurate predictions remains a challenge. To enable the proper strategies for both prevention and timely control, more variables with potential impact, including environmental factors (e.g. climate variables) and socioeconomic factors should be considered in further studies to increase the predictive power of the model, because HFMD is a complex communicable disease [30, 31].

One limitation of this study is due to the assumption that HFMD epidemics were influenced by latitude variation and followed a spatial spread pattern from the south to the north. It is possible that our results will not generalize to other infectious diseases without such a spatiotemporal characteristic. The objective of this study, however, was to explore the influence of the variation in latitude on HFMD epidemics. It is possible that using a large amount of surveillance data and taking into account more characteristics of the studied infectious disease could further improve detection performance.

The use of a regression approach may induce another limitation of this paper. Regression analysis is widely used for aberration detection and prediction, but regression modeling generally requires a considerable amount of data to provide stable parameter estimates. In some areas such as a designated area located at or near the southernmost or the northernmost part of a geographic range (e.g. Okinawa and Hokkaido in Japan), it may not be feasible to monitor HFMD epidemics by using the proposed approach. For such areas, time series models, such as SARIMA models, may be utilized for interpreting and applying the HFMD surveillance data for disease control and prevention.

Due to a reporting hierarchy of public health systems, there is an inherent reporting lag in sentinel surveillance data. The time lag between disease onset and the date of report publication was up to one week in Japan. We know the timeliness is a key performance measure of public health surveillance systems. However, the timeliness can vary by disease, intended use of the data, and public health system level. The incubation period of HFMD is 3 to 5 days (with a range from 2 days to 2 weeks). In Japan, the time lapse between onset date and the date of report was short enough to initiate preventive measures and provide early health warnings to the public. In Hong Kong, Malaysia, Japan, the Republic of Korea and Singapore, sentinel surveillance systems have been implemented to monitor HFMD epidemics on weekly basis in order to allow the health authorities to issue early warning of seasonal activity, detect abnormal aberrations and assess the impact of public health control measures. Current sentinel reporting timeliness in Japan may be sufficient to support an immediate public health response in the event of an HFMD epidemic.

Finally, HFMD is caused by several enteroviruses. In this study, there was insufficient information on causal agents, for example, on whether enterovirus 71 (EV71) or coxsackievirus A16 (CVA16) was responsible for the epidemics in Japan during the study period. This may have affected the disease duration or epidemic peaks [33]. In addition, the HFMD case identification depended mainly on clinical presentation, without confirming the diagnosis by microbiological or serological tests, hence resulting in potential misdiagnosis. However, HFMD is considered to be an easily recognized disease by pediatricians [16, 34, 35].

## Conclusions

The frequency and scale of HFMD outbreaks are expected to increase [3, 12], and threaten the health security of various nations due to continuing viral mutation [3], climate change [1, 23], and the lack of health resources and effective surveillance systems in some countries [3]. A reliable early warning model can help public health agencies to take preventive actions to control HFMD epidemics at an early stage, thus reducing their impact on the health system and society.

This paper first attempts to explore the influence of latitude variation on HFMD epidemics. We have used only the latitude, one spatiotemporal feature of HFMD, to develop rules for early aberration detection and prediction. We have also demonstrated that the proposed rules performed well on real data in terms of accuracy and timeliness. Although our approach may provide helpful information for controlling epidemics and minimizing the impact of diseases, the performance could be further improved by including other influential meteorological factors along with the proposed latitude-based approach, which is worth further investigation.
